# Antifungal Resistance in Cryptococcal Infections

**DOI:** 10.3390/pathogens13020128

**Published:** 2024-01-29

**Authors:** Marcia S. C. Melhem, Diniz Pereira Leite Júnior, Juliana P. F. Takahashi, Milena Bronze Macioni, Lidiane de Oliveira, Lisandra Siufi de Araújo, Wellington S. Fava, Lucas X. Bonfietti, Anamaria M. M. Paniago, James Venturini, Ana Espinel-Ingroff

**Affiliations:** 1 Graduate Program in Sciences, Secretary of Health, São Paulo 01246-002, SP, Brazil; djbiologico@gmail.com (D.P.L.J.); milenabmacioni@gmail.com (M.B.M.); 2 Graduate Program in Infectious and Parasitic Diseases, Federal University of Mato Grosso do Sul, Campo Grande 79070-900, MS, Brazil; julianaptakahashi@gmail.com (J.P.F.T.); wegfava@gmail.com (W.S.F.); anapaniago@yahoo.com.br (A.M.M.P.); 3 Graduate Program in Tropical Diseases, State University of São Paulo, Botucatu 18618-687, SP, Brazil; 4 Pathology Division, Adolfo Lutz Institute, São Paulo 01246-002, SP, Brazil; 5 Grupo Fleury Laboratories, São Paulo 04701-200, SP, Brazil; oliveira.lde@hotmail.com; 6 Central Public Health Laboratory-LACEN, Mycology Unit, Adolfo Lutz Institut, São Paulo 01246-002, SP, Brazil; lucasxbonfietti@yahoo.com.br; 7 Central Public Health Laboratory-LACEN, Campo Grande 79074-460, MS, Brazil; victoria.ingroff@vcuhealth.org; 8 VCU Medical Center, Richmond, VA 23284, USA

**Keywords:** fluconazole, amphotericin B, resistance, azoles, CLSI, EUCAST ECVs

## Abstract

Antifungal therapy, especially with the azoles, could promote the incidence of less susceptible isolates of *Cryptococcus neoformans* and *C. gattii* species complexes (SC), mostly in developing countries. Given that these species affect mostly the immunocompromised host, the infections are severe and difficult to treat. This review encompasses the following topics: 1. infecting species and their virulence, 2. treatment, 3. antifungal susceptibility methods and available categorical endpoints, 4. genetic mechanisms of resistance, 5. clinical resistance, 6. fluconazole minimal inhibitory concentrations (MICs), clinical outcome, 7. environmental influences, and 8. the relevance of host factors, including pharmacokinetic/pharmacodynamic (PK/PD) parameters, in predicting the clinical outcome to therapy. As of now, epidemiologic cutoff endpoints (ECVs/ECOFFs) are the most reliable antifungal resistance detectors for these species, as only one clinical breakpoint (amphotericin B and *C. neoformans* VNI) is available.

## 1. Introduction

Fungi cause a wide range of life-threatening infections, especially among immunocompromised individuals (~278,000 infections and 180,000 deaths/year) [1]. Cryptococcosis frequently presents lung lesions; these can evolve into meningoencephalitis, fatal pulmonary disease meningitis, or disseminated cryptococcosis among AIDS patients and, lately, in patients with severe COVID-19 infections. These patients require prompt and adequate antifungal therapy [2,3]. Cryptococcal infections are also associated with the extensive use of corticoids among organ transplant patients (e.g., CD4 T cell counts of <200 cells/mm^3^). Rajasingham et al. [4] estimated that approximately 4.3 million adults with HIV have CD4 counts below 200 cells/mm^3^. Cryptococcosis is caused by the *Cryptococcus neoformans* and *C. gattii* species complexes (SC). While *C. gattii* primarily causes pulmonary disease, meningitis is mostly associated with *C. neoformans* [5,6,7]. Among severely immunosuppressed HIV patients, *C. neoformans* infections are more frequent than those involving *C. gattii* [8,9]. However, *C. gattii* has also been isolated from immunocompetent humans and animals (a historic epidemic on Vancouver Island, Canada) [10]. In recent years, we have benefited from knowledge gained on the genetic, physiological, pathogenic, and ecological features of *Cryptococcus* SC [11,12,13]. Therefore, this review encompasses the following topics: 1. infecting species and their virulence; 2. antifungal treatments; 3. antifungal susceptibility testing methods and available categorical endpoints; 4. genetic mechanisms of resistance; 5. clinical resistance; 6. fluconazole’s minimal inhibitory concentrations (MICs) and clinical outcomes; 7. environmental influences; and 8. the relevance of host factors, including pharmacokinetic/pharmacodynamic (PK/PD) parameters, in predicting the clinical outcome of therapy.

## 2. Cryptococcal Genotypes: Virulence Factors

Based on their genotypic diversity, the *C. neoformans*/*C. gattii* species complexes can be separated into seven species [13] harboring distinct virulence factors, beyond antifungal resistance profiles, that have an impact on refractory and chronic cryptococcal infections [14,15]. *Cryptococcus* genotypes and virulence were largely studied in the Vancouver Island outbreak, and they were well correlated with certain molecular types of *C. gattii* [10,16]. The in vitro and in vivo data revealed that the VGIIa subtype is more virulent than the VGIIb subtype [16]. Higher virulence of clinical isolates of both subgenotypes compared to environmental isolates showed that clinical isolates were more virulent than environmental isolates using an mice nasal inhalation model [16]. Among the *C. neoformans* SC, the VNI genotype is associated with microcells (diameter < 1 µm), which can facilitate dissemination to the central nervous system, contributing to the occurrence of the severe meningoencephalitis form of the disease [17].

It is of clinical relevance that, in some patients, the coexistence of different phenotypes and/or genotypes has been demonstrated, and infections caused by multiple *C. neoformans* genotypes show different resistance patterns during and/or after antifungal treatment [18,19,20,21,22,23]. At least 208 studies have described the occurrence of infections due to mixed *C. neoformans* genotypes and their competitive status in the host. In routine laboratory testing, the scenario of multiple-strain infection within the host is rarely considered. So, when a single *C. neoformans* colony is used for testing, mixed infections are potentially missed, and it is more likely that only one genotype is evaluated [22].

Certain *C. neoformans* genotypes may become more prevalent under the selective pressure exerted by antifungal therapy [23]. The Mechanisms of Resistance section below discusses some of these issues. There are reports of patients having recurrent infections and possibly harboring two or three genetically identical isolates, while other patients may have relapsed with genetically different isolates [18,19,20,21,22,23]. Relapse may or may not necessarily be associated with changes in the in vitro antifungal susceptibility pattern [23]. An isolate that persists over time acquires phenotypic diversity regarding its susceptibility to phagocytic cells, gaining capacity for cytokine responses or surface adhesion, without any alteration of the genotype, or by instability and microevolution of the karyotype [19,22]. For example, the physiological properties of *C. neoformans* regarding its amino acid uptake system, an important factor for virulence, showed the need for amino acid permeases (encoded by *Aap4*/*Aap5*) during resistance to thermal and oxidative stress in the process of invasion and host colonization [24].

Important pathogen factors during infection include the polysaccharide capsule size [25,26,27] and melanization, which directly modulates virulence by evading the host’s immune system [28,29]. Other virulence factors include extracellular enzymes, such as laccases and phospholipases, 37 °C thermotolerance, the presence of filamentous forms, the ion uptake profile, and/or the production of titan cells [30,31,32]. Additionally, the fungal wall plays a role in virulence, since the spatial organization and dynamic regulation change the properties of hydrophobicity, adhesiveness, and chemical and immunological heterogeneity in response to environmental growth conditions [33].

Some laboratory data are available on the interplay of virulence properties and genotypes, contributing to our understanding of their pathogenesis; for example, it was described that the VNII lineage displays increased laccase activity [34]. However, few studies have addressed the correlation between the phenotype of virulence and clinical presentation. Evidence of the genetic lineage’s association with phenotype and cryptococcosis clinical outcomes was first provided in a Ugandan study [35]. The effect of fungal genotypes on clinical outcomes was also observed in Southern African patients, with those infected with the VNB lineage having significantly worse survival in comparison to those infected with VNI or VNII genotypes [34]. A comprehensive review summarizing the current understanding of how *C. neoformans* genotypes and phenotypes modulate patient outcomes and recurrence was conducted by Altamirano et al., 2020 [36]. Otherwise, the mechanisms of how genotypes and phenotypes contribute to clinical outcomes remain unknown, probably due to the genetic heterogeneity of *Cryptococcus* SC and the extensive phenotypic variation observed between and within infective strains.

## 3. Fungal Treatment Challenges

The main life-threatening forms of cryptococcosis are cryptococcal meningoencephalitis and disseminated disease. No effective cryptococcal vaccines are available to avoid this disease [37], and the management of cryptococcal meningitis is restricted to a limited drug armamentarium. Three antifungal drugs have reliable efficacy in vivo: amphotericin B, flucytosine, and fluconazole. The therapeutic regimens are long, and these agents are significantly toxic, especially amphotericin B [38].

Cryptococcal meningitis antifungal treatment for AIDS patients occurs in three phases: induction, consolidation, and maintenance. Amphotericin B plus flucytosine is recommended for the induction stage, while fluconazole is recommended for the consolidation and maintenance phase [39]; fluconazole is also considered for the induction stage when amphotericin B is inaccessible. Flucytosine, a drug that rapidly induces resistance, is not recommended for administration alone but is recommended in combination therapy with amphotericin B [40].

Although clinical microbiological resistance to antifungal therapy has not been a major problem in disease management, the response rate to therapy in AIDS-related cryptococcosis is low, and the fatality rate is as high as 40% at 10 weeks [40]. 

One host factor that determines clinical resistance is the site of the infection. As the central nervous system (CNS) is the most affected site in cryptococcosis, the antifungal concentration in the brain tissue or cerebrospinal fluid (CSF) can influence the therapeutic response. Fluconazole and flucytosine penetrate relatively well into both, while the itraconazole concentration in CSF is very low [41]. Amphotericin B, as deoxycholate or in a lipidic formulation, achieves similar levels in the brain and cerebrospinal fluid, but the tissue or cerebrospinal fluid/plasma concentration ratio has been demonstrated to be low [41]. The concentration–time area under the curve (AUC) values relative to the minimal inhibitory concentration (MIC) of the antifungal agents and their association with clinical outcomes in cryptococcal infection indicate the clinical breakpoint and are discussed in a later section. The prostate has been considered a persistent focus of *Cryptococcus* in patients under or after antifungal treatment [42,43] and a critical organ responsible for relapses; there is little evidence of a low fluconazole concentration in human prostate tissue [44,45].

Fluconazole and amphotericin B affect the structure of the cell membrane as their main mechanism of action [46,47,48]. Fluconazole, like other azoles, acts during the formation process of ergosterol, an important structural lipid. Amphotericin B removes ergosterol from the cytoplasmic membrane, promoting the formation of pores and channels, causing instability of the cell’s permeability and subsequent cell death. However, it also causes oxidative cell stress, interrupting the host’s immune responses and consequently allowing the fungal cells to spread [49,50]. Treatment success in invasive cryptococcosis depends on a combination of factors related to the pathogen, the host, and the antifungal agent, and understanding the stress and adaptation of the agent in the host is crucial. The mechanisms involved are diverse and not yet fully understood.

## 4. Antifungal Susceptibility Testing Methods and Categorization Endpoints

An important role of antifungal susceptibility testing is to detect in vitro resistance, especially when a patient is not responding to therapy. The breakpoint (BP) is the best in vitro resistance indicator, but Clinical Laboratory Standards Institute (CLSI) BP values are not available for *Cryptococcus* SC. The European Committee on Antimicrobial Susceptibility Testing (EUCAST) has proposed an amphotericin BP vs. *C. neoformans* VNI [51]. On the other hand, the CLSI has defined epidemiological cutoff values (ECVs) [52], another categorization endpoint, for amphotericin B, fluconazole, itraconazole, voriconazole, and flucytosine vs. the main molecular types of C. neoformans SC or C. gattii SC [53]. These CLSI ECVs can be used to categorize an isolate as a non-wild type (NWT or mutant). EUCAST has also proposed epidemiologic cutoff values, named ECOFFs, for posaconazole and amphotericin B against *C. neoformans* VNI, in addition to ECOFFs for amphotericin B and posaconazole against *C. gattii VGI* [51] (Table 1).

The CLSI-stipulated fluconazole MIC of >16 g/mL for *C. neoformans* is predictive of clinical failure [54,55,56,57,58,59]. The establishment of BPs demands available clinical outcome data, in vitro and animal experiments, dosing information, pharmacodynamic/pharmacokinetic parameters, and MIC distributions [52]. One of the problems is the lack of clinical data. The other problem is the potential lack of reliability of the in vitro results [60,61,62].

Due to the complexity of both reference methodologies, they are not the best tools for use in small laboratories; however, some commercial methods are also available (e.g., YeastOne, Etest, Neo-Sensitabs, and others) for testing *Cryptococcus* SC [63,64]. Ideally, the results should be confirmed using a reference method, especially when ECVs or BPs have been estimated for these species using the commercial methods.

## 5. Mechanisms of Resistance

The exacerbated resistance observed in some pathogens, e.g., *Candida* or *Aspergillus,* to almost all clinically approved antifungal agents is not observed among *Cryptococcus* isolates, with only fluconazole being problematic in this regard; amphotericin B still serves as the gold-standard treatment for systemic fungal infections, with minimal development of clinically important resistance [49]. The emergence of resistance to flucytosine prevents its use as a monotherapy drug for cryptococcosis antifungal treatment, so it is used in combination with amphotericin B as a first-line induction treatment, as cited previously [40]. The process leading to the emergence of fluconazole resistance in *Cryptococcus* SC is challenging to identify, and many studies are dedicated to elucidating it [65].

Antifungal resistance involves several different mechanisms: the occurrence of mutations in the *erg11* gene of the ergosterol biosynthesis pathway, stress signaling, efflux pumps, membrane traffic, genetic modifications, and aneuploidies [66,67]. Most of the genetic causes of azole resistance involve *erg11* gene mutations resulting in amino acid substitutions in the 14 alpha-demethylase enzyme, which is the azole drugs’ target [66]. These mutations can affect the affinity of azole drugs to the enzyme, causing a loss of antifungal activity [66].

Sanguinetti et al. [68] demonstrated that the gene encoding of the ATP binding cassette (ABC) transporter (“*C. neoformans* AntiFungal Resistance 1—cnAFR1”) is involved in active fluconazole efflux, leading to in vivo resistance in *C. neoformans*, and plays a role in the enhancement of cryptococcal virulence. As cryptococcal isolates have evolved from *Filobasidiella*, which encodes the gene AFR, involved in in vitro fluconazole resistance, it is likely that this phenomenon is an azole-inherent mechanism within both species [69]. The ABC transporters have a broader specificity, so they could cause multidrug resistance, while AFR1 is the azoles’ main efflux pump for these species [70,71,72]. As recently reported, the AFR3-ABC transporter (CNAG_06909), which is expressed in *C. neoformans* at an advanced generational age, could become cumulative during a chronic infection [71]. The *Cryptococcus* genome contains a particular gene in the cell plasma membrane and the endoplasmic reticulum called ABC PDR6 (CNAG_06909); this gene influences the synthesis of ergosterol and acts as a regulator of phagocytosis. The atypical transporter, unknown until recently, is highly conserved and is associated with fungal multidrug resistance and increased pathogenesis [71]. The novel ABC pump may contribute to enhanced fluconazole resistance by promoting drug efflux, and it promotes macrophage resistance, contributing to the selection of older *C. neoformans* cells during chronic infection [71].

Gerstein et al. [73] indicated that during therapy, the emerging fungal cells—polyploid titan cells—generate daughter cells that exhibit increased resistance to fluconazole, adapting to the host environment. The cell wall [25] and capsular structure [74] have structural differences in titan cells compared to the canonical yeast cells; titan cells are more resistant, and they can promote cell protection against oxidative stress and phagocytosis [75,76], disturbing the host’s immune responses [76,77]. This gigantism leads to fungal persistence in the host and could cause disease relapses [76]. Thus, the titan cells are a mechanism for generating genomic plasticity and aneuploidy, leading to resistant *C. neoformans* isolates [78].

In addition to contributing to fluconazole resistance through titanization, aneuploidy also plays a role in the development of drug heteroresistance. Fluconazole heteroresistance, demonstrated in *C. neoformans* and *C. gattii*, refers to the presence of a certain cell subpopulation (10–14% of the total population) with inherent antifungal resistance that is reversible; this occurs regardless of drug exposure, confirming that the phenomenon is adaptive [79,80,81,82,83,84,85]. Sionov et al. (2009) [79] found heteroresistance to fluconazole in clinical and environmental strains of *C. neoformans* from 1979 onwards, and they observed that strains isolated from patients with HIV/AIDS presented higher levels of the phenomenon, causing a 16-fold increase in the MIC value after exposure to the drug. A higher proportion of *C. gattii* strains (86%) than *C. neoformans* (46%) was observed among 71 clinical and environmental strains, confirming heteroresistance as an intrinsic mechanism associated with the virulence of the strain that may be linked to stress suffered in the environment [80]. Genomic analyses have shown that this heteroresistance is mediated by the aneuploidy of chromosomes 1, 4, 6, and 10, with chromosome 4 (Chr4) being the second most frequently found to be disomic, after Chr1, in resistant subpopulations (named heteroresistant clones). *C. neoformans* Chr4 does contain two ABC transporters and a homolog of PDR16, suggesting the relevance of chromosomal duplication for the agent’s survival at high concentrations of fluconazole as a strategy for drug resistance [86]. The phenomenon of heteroresistance was recently associated with therapeutic failure in the antifungal treatment of cryptococcosis in patients showing increasing aneuploid populations of *C. neoformans* over the course of fluconazole monotherapy [87].

## 6. Clinical Resistance

In spite of the overall rarity of amphotericin-B-resistant strains and low rates of fluconazole in vitro resistance, relapse and/or refractory illness, as well as high mortality, are common features of cryptococcal infections; therefore, a successful outcome depends on several factors besides the antifungal susceptibility of the causative agent [88,89,90]. As mentioned before, the antifungal concentration in brain tissue or cerebrospinal fluid (CSF) differs among drugs and impacts the therapeutic response [41]. Poor clinical outcome has been reported in association with intracranial hypertension [91], high fungal burden [91,92,93], slow rates of CSF sterilization [94], and altered mental status on admission [95].

In addition to the drug susceptibility of the causative strain, resistance could occur due to an alteration of the morphological structure of the cell wall, where the exopolysaccharide capsule becomes thicker and contributes to antifungal resistance and the formation of titan cells, as described before [77,78].

Other common complications are adverse antifungal effects, including renal dysfunction, anemia, electrolyte disturbances, and infusion site reactions, especially with amphotericin B therapy; gastrointestinal intolerance presenting as abdominal pain, elevated aminotransferases, and bone marrow suppression by flucytosine; or even rash and liver enzyme abnormalities, especially with high doses of fluconazole, which may lead to drug discontinuation or treatment failure [96,97,98].

Many factors affect the antifungal treatment response to fungal infection, and among these, resistance to antifungal agents is likely a factor that leads to the failure of cryptococcosis treatment; thus, an evaluation of the treatment agent’s antifungal susceptibility is important since high values have been reported, especially with fluconazole treatment [54,99,100]. Conversely, resistance to fluconazole prior to therapy was not considered a major clinical problem [101,102].

High MICs for distinct clinical *Cryptococcus* genotypes have been reported from a variety of countries [103,104]. In population-based surveillance (2002–2003 and 2007–2008) of *C. neoformans*, the fluconazole and voriconazole MICs were ≥16 mg/L (2001 to 2011) prior to azole therapy only for 0.6% of 487 incident isolates [105].

Species complexes and virulence also contribute to clinical resistance, and although the incidence of *C. gattii* infections is lower than that of *C. neoformans*, the following characteristics are a concern [106,107]: *C. gattii* is generally less susceptible to antifungal agents [108,109], and some VGII strains are less susceptible to azoles, especially the Vancouver Island strains, which are also recognized as hypervirulent compared to those from other outbreaks [16,109]. Therefore, patients with *C. gattii* infections could have a slower or incomplete response to antifungal therapy; such patients also often have neurological sequelae that require surgery and prolonged therapy [107,108]. On the other hand, fluconazole MICs for the VNII genotype could be lower than those for other genotypes; consequently, molecular characterization is also important [109,110].

However, due to the lack of fluconazole BP values, isolates can only be classified by means of ECV/ECOFF as either mutant (NWT, which could be more difficult to treat) or WT, which is not the same as resistant or susceptible [52]. In the clinical setting, the concern should be what method and classification endpoints are available to evaluate the infecting *Cryptococcus* isolate [62,111].

The mortality rate among patients with *C. neoformans* variety *neoformans* (VNI, VNII, or VNIII) infection is usually high. Patients with *C. gattii* SC often have neurological sequelae that require surgery and prolonged therapy. It is notable that in some of these reports, no in vitro versus in vivo correlation was reported [101,112].

**Table 1:** Species complexes of the genus *Cryptococcus* and available ECVs (CLSI) or ECOFFs (EUCAST) for several antifungal agents.

Antifungal Agent	Proposed Species (Genotype)	CLSI ECV ^1,2,3^ (mg/L)	EUCAST ECOFF or BPs ^4^ (mg/L)		
		WT ≤	WT ≤	S ≤	R >
Amphotericin B	*C. gattii* (VGI)	0.5	0.5	-	-
	*C. deuterogattii* (VGII)	1	-	-	-
	*C. neoformans* (VNI)	0.5	1	1	1
Flucytosine	*C. gattii* (VGI)	4	-	-	-
	*C. deuterogattii* (VGII)	32	-	-	-
	*C. neoformans* (VNI)	8	-	-	-
Fluconazole	*C. gattii* (VGI)	16	-	-	-
	*C. deuterogattii* (VGII)	32	-	-	-
	*C. neoformans* (VNI)	8	-	-	-
Itraconazole	*C. gattii* (VGI)	0.5	-	-	-
	*C. deuterogattii* (VGII)	1	-	-	-
	*C. neoformans* (VNI)	0.25	-	-	-
Posaconazole	*C. gattii* (VGI)	-	1	-	-
	*C. deuterogattii* (VGII)	-	-	-	-
	*C. neoformans* (VNI)	0.25	0.5	-	-
Voriconazole	*C. gattii* (VGI)	0.5	-	-	-
	*C. deuterogattii* (VGII)	0.5	-	-	-
	*C. neoformans* (VNI)	0.25		-	-

CLSI (Clinical and Laboratory Standards Institute); EUCAST (European Committee on Antimicrobial Susceptibility Testing); ECVs and ECOFFs (epidemiologic cutoff values); BPs (clinical breakpoints); WT (wild type); S (susceptible); R (resistant); VG (molecular type of *C. gattii*); VN (molecular tpe of *C. neoformans*); ^1^ CLSI, M59S. document (2022) [53]; ^2^ Espinel-Ingroff et al., 2012 [103]; ^3^ Espinel-Ingroff et al., 2012 [104]; ^4^ EUCAST (2023) [51].

## 7. Clinical Outcome: The Role of Antifungal Susceptibility Testing

### 7.1. Amphotericin B Alone or as Part of Combined Therapy

As mentioned in Item 4 (Antifungal Susceptibility Testing Methods and Categorization Endpoints), clinical practice would greatly benefit if fluconazole and amphotericin B susceptibilities could be correctly interpreted or classified using BP values. The CLSI and EUCAST methods are available for antifungal susceptibility testing of *Cryptococcus* SC and other fungal species [113,114]. In addition, strip diffusion tests and custom multi-well plates (YeastOne Sensititre^®^, SYO) are also available, but without categorical endpoints [63,64,88]. Therefore, ECVs for the commercial methods and BPs for the reference assays are needed. EUCAST has proposed an amphotericin B BP (1 mg/L) for *C. neoformans* SC, but nothing has been reported about further establishment of other endpoints for this fungal group [51]. The first case of an amphotericin B failure was reported for an AIDS patient with a *C. neoformans* infection (amphotericin B MIC of 4 mg/L via the microdilution method) [115]. However, such a result is rare [67]. A large series of patients from Kampala, Uganda, was evaluated (1998–1999 and 2010–2014); only eight amphotericin B MICs were >1 mg/L (17%), but the clinical outcome was poor for the combined amphotericin B and fluconazole therapy [116]. Two studies, using a modified quantitative broth microdilution susceptibility method, reported a strong association between values and patient outcome (<15 cells in the CSF and survival by day 14 of therapy). The first study evaluated 85 patients, and the second evaluated 15 patients [42,117].

Mortality due to cryptococcosis among patients co-infected with HIV and treated with amphotericin B and fluconazole was not associated with MIC data, indicating low resistance rates from 0% to 5.8% for *C. neoformans* and *C. gattii*, respectively [60,101,118,119,120,121,122,123,124,125,126,127,128]. Therefore, in general, we need a better way to evaluate the in vitro correlation with clinical response to therapy [62,121,129].

### 7.2. Fluconazole: In Vitro and Clinical Data

Some studies have demonstrated no association between the in vitro susceptibility of the infecting *Cryptococcus* isolate and clinical outcomes. Even infected patients with low fluconazole MICs (<16 mg/L) may suffer therapeutic failure [55,60,93,101], with no evidence that antifungal susceptibility testing can help guide antifungal treatment in cryptococcal meningitis cases.

The relationship between fluconazole in vitro data and clinical response is not always clear [129,130,131,132,133,134,135,136,137]. Over 4% of 143 AIDS patients relapsed during prolonged fluconazole therapy; there was an 8- to 12-fold increase in the CLSI or SYO fluconazole MICs for serial isolates (up to 5 months) [129]. Fluconazole clinical failure during *C. neoformans* infections correlated with the in vitro data of the infecting isolates (MICs of ≥64 mg/L) [133]; these patients had a positive cryptococcal antigen and a CD4 count of <100 cells/μL. An approximately 15% fluconazole failure rate versus MICs up to ≥128 mg/L was also reported [133,134,135,136,138,139]. However, other data indicated that fluconazole MICs for cryptococcal isolates correlated with a response to therapy [54,61,136,140] or that values of >8 mg/L versus clinical response were ambiguous given that the CLSI ECV was ≤16 mg/L. In other studies, the conclusion was that there was no correlation between CLSI in vitro data and the fluconazole treatment outcome [55,60,89,90,101,130].

There is also a question as to whether the CLSI method was properly performed to allow proper comparisons between the literature findings. The problem is that partial current data are given by the commercial methods, and as mentioned before, ECVs are available for these methods for other yeast species, but not for *C. neoformans*. If laboratories are using a commercial method, the isolate should be sent to a reference center for corroboration. Commercial companies should also try to develop ECVs.

Lastly, we should mention the “90–60 rule”, which refers to a pattern observed in therapeutic outcomes concerning infections and their responses to specific antifungal treatments based on their susceptibility to antimicrobial agents [141]. Broadly, this rule states that infections caused by susceptible isolates respond positively to appropriate therapy around 90% of the time. In contrast, infections caused by resistant isolates (or those treated with inappropriate drugs) respond to treatment approximately 60% of the time. This rule implies a correlation between the susceptibility of the infectious agent to antibiotics and the effectiveness of the treatment provided. It indicates that infections caused by microbes susceptible to the prescribed antibiotic regimen tend to respond more favorably compared to those caused by resistant strains or those treated with inappropriate antibiotics. These observations relate specifically to immunocompetent patients with monomicrobial infections receiving a single antimicrobial agent administered intravenously under circumstances where penetration of the drug to the site of infection is predictable [141]. These conditions do not account for the majority of cryptococcosis patients, who could present polygenotype infections, be treated with multiple antifungals, receive oral fluconazole, or have compromised immune systems. In these diverse situations, how reliable are antimicrobial susceptibility test results in guiding clinical decisions? Their reliability is significantly lower. In fact, in many of these scenarios, in vitro susceptibility test results have little to no prognostic value.

## 8. Environmental versus Clinical Isolates

The genus *Cryptococcus* is widely distributed in nature, having been present in soil samples, bird nests, urban and wild bird droppings, hollow trees, plant remains, organic materials, and decaying wood [142,143,144]. Comparisons of in vitro data for clinical versus environmental isolates are scarce [69,122,145,146,147]. There are few reports of environmental isolates that are susceptible to antifungal drugs, including fluconazole, or describing higher resistance in clinical isolates in comparison to the environmental ones [145,146,147,148,149]. In general, environmental isolates are less susceptible to fluconazole among the agents used, in comparison to the clinical isolates (e.g., fluconazole: 0.25–64 mg/L vs. 0.25–8 mg/L); this tendency was particularly observed with VNI strains from the environment. Similar findings have also been reported from Poland, Thailand, India, Cuba, and other areas [143,144,145,146,147,148,149,150,151,152,153]. These data point out the occurrence of primary resistance among environmental isolates of both *C. neoformans* and *C. gattii.* Low susceptibility profiles could be due to environmental temperature changes, a poor nutrient supply, or oxidative factors [154,155]. Environmental factors driving cross-resistance to triazole drugs are most likely due to the greater use of azole fungicides and agrochemical products exerting selective pressure on isolates in nature [156,157,158]. These findings highlight the impact of using azole fungicides, leading to an environmental route of resistance that could negatively influence fluconazole monotherapy for *C. neoformans* infections [159].

## 9. Role of PK/PD Levels

Patients’ drug levels and MIC results are important [160]. Fluconazole plasma levels are generally higher than those of itraconazole [161]. The antifungal susceptibility of an isolate is based on the pharmacokinetic (PK) and pharmacodynamic (PD) drug concentrations or levels and their effects during antifungal therapy. PK–PD ratios have been defined in experimental studies and could be predictive of therapeutic outcomes. However, on a fixed regimen of antifungal therapy, PK results are variable, and such levels can be quantified by population PK modeling. Monte Carlo simulation is a useful tool in replicating pharmacokinetic variability [162]. For example, the role of fluconazole is to inhibit growth, not to kill. In general, this activity is due to the emergence of cryptococcal resistance, which frequently cannot be detected by means of the available standard methods. This resistance may be potentially overcome by dosage escalation or the use of combination therapy, e.g., amphotericin B plus flucytosine, which could improve survival among patients with cryptococcal meningitis. However, the use of amphotericin B plus fluconazole was not found to correlate with an improvement in survival [163].

The strongest relationship between fluconazole PK levels and clinical outcomes of therapy in patients with cryptococcal meningitis seems to be for the PK–PD index AUC/MIC. The AUC is the area under the drug curve. The target fluconazole concentration in body fluids during cryptococcal infections has not yet been defined; the same applies to the exact target AUC/MIC ratio for fluconazole consolidation or prophylaxis [164].

Recently, an arbitrary AUC/MIC ratio of >100 was employed to determine the probability of success in fluconazole monotherapy at distinct doses. A systematic review of data records of the fluconazole MIC distribution against clinical *Cryptococcus* isolates was used to compare the plasma fluconazole concentrations of African patients. Fluconazole at a dose of 800 mg/day or 1200 mg/day could be a more effective choice, with uptrending MICs [164].

## 10. Perspectives

As shown in this literature review, there are still several gaps in the detection of antifungal resistance for cryptococcosis management. We believe that the correlation of in vitro antifungal data and therapy response is inadequate in the clinical setting. There are no easy diagnostic methods to detect the molecular mechanisms of antifungal resistance among *Cryptococcus* SC. The available commercial and reference methods lack sufficient categorical endpoints due to the lack of in vivo data from clinical trials. It is now evident that more than one colony should be evaluated to reveal potential co-infections (e.g., multiple genotypes) and properly assess the susceptibility of the etiologic agent.

The association of the MIC with the specific antifungal PK–PD target could provide important information on whether the patient is likely to benefit from antifungal therapy. This information is also needed for establishing species–method–agent BPs to better manage cryptococcosis and other fungal infections.

We highlight the importance of constant network surveillance of the emergence of resistant species among both environmental and clinical isolates, especially during therapeutic failure or for those patients receiving prolonged antifungal therapy. Perhaps a public health advocacy strategy is essential to educate and organize health personnel and authorities. Until then, the correlation of MICs and clinical outcomes of cryptococcal diseases remains inadequate.

## Data Availability

Not applicable.
